# Specifications for Innovative, Enabling Biomaterials Based on the Principles of Biocompatibility Mechanisms

**DOI:** 10.3389/fbioe.2019.00255

**Published:** 2019-10-09

**Authors:** David F. Williams

**Affiliations:** ^1^Wake Forest Institute of Regenerative Medicine, Winston-Salem, NC, United States; ^2^Strait Access Technologies, Cape Town, South Africa

**Keywords:** host response, inflammation, mechanotransduction, implant, template

## Abstract

In any engineering discipline, whenever products are designed, manufactured and ultimately utilized for the benefits of society, a series of specifications for the product are defined, and maybe refined, in order that they perform as effectively as possible, with due attention being paid to the safety, and economic aspects. These specifications are established with respect to all of the relevant properties, including those determined by mechanical, physical, chemical, manufacturing and environmental conditions, and the resulting design and materials selection reflects the optimal balance. In areas of medical technology, these specifications should be based on both functionality, which determines whether a device can actually perform as intended, and biocompatibility, which determines how the device interacts, both acutely and chronically, with the body. Unfortunately, whilst so much progress has been made with the development of superior functionality for the treatment and diagnosis of so many disease states, this is not the same for biocompatibility, where the single most-important currently adopted specification is that the device should do no harm, which falls far short of the ideal requirement. This paper addresses the profound need for biomaterials specifications to be based on the mechanisms of biocompatibility.

## Introduction

We have recently re-defined the term biomaterial as “*a material designed to take a form that can direct, through interactions with living systems, the course of any therapeutic or diagnostic procedure*” (Zhang and Williams, 2018). The two critical parts of this definition relate to the objectives of the systems in which a biomaterial is used and the fact that the material has to interact with living systems, in most cases parts of the human body, in order for these objectives to be realized. This definition, and indeed, the whole concept of biomaterials science, applies equally to situations involving implantable devices, artificial organs, tissue engineering templates, non-viral gene vectors, drug delivery systems and contrast agents. When determining the specifications for the biomaterials used in every application, it is natural that the functional requirements are considered first; after all, there is no point in using an opaque material for an intraocular lens, a rigid metallic scaffold for tissue-engineered cartilage or an unresponsive elastomer for an MRI contrast agent. In the majority of situations, there are predicates that give some idea of the functional characteristics that are likely to be appropriate, and a variety of laboratory and pre-clinical tests allow designers and manufacturers to refine materials selection parameters and, hopefully, optimize the final choice. These procedures are facilitated by lists of materials known to have previously received regulatory approval in similar types of situations, and international standards that advise on the tests that could or should be performed.

So far, so good. But what about the ability to control the function through interactions with living systems. These interactions are generally considered within the phenomena related to biocompatibility. This term has also been recently reconsidered (Zhang and Williams, 2018), when the original definition agreed in 1986 (Williams, 1986), was confirmed as still correct, being “*the ability of a material to perform with an appropriate host response in a specific application*.”This is important since it mandates that the performance of a biomaterial is dependent on the host response, as now indicated in the definition of biomaterial, and especially that this response will vary from one application to another. This is an excellent contextual definition but it does not, indeed cannot, tell us how to design a material with good, or even appropriate, biocompatibility.

The main problem with this situation, as implied with reference to the dependence of biocompatibility on the application, is that biocompatibility is not a property of a biomaterial, but of a biomaterial-host system. As emphasized in several recent publications, the biocompatibility characteristics of a material will vary depending on specific biological and clinical factors. No material can be described as a generic “biocompatible” material (Williams, 2008; Williams and Williams, 2014). It is unfortunate that even today, major journals include papers that refer to biocompatible materials, as do documents from the FDA and other regulators, and also those of the most widely used medical device standards.

The theme of the present paper is that in order to design better biomaterials for future clinical therapies, we need to identify specifications for biocompatibility as well as functionality, and this will have to take into account the need to define the precise material-host system and not just the material. The discussion will focus on the pathways involved in the host response, using three scenarios within implantable devices, tissue engineering, and contrast agents to reinforce the arguments.

## Biocompatibility and Toxicity

For a long time, and indeed before the term biocompatibility became recognized, the ideal characteristic of a biomaterial was considered to involve a lack of any effect on the body (Williams and Roaf, 1973; Scales and Winter, 1975), often couched in terms of having no adverse effect, but in reality this equated with having no effect on, or no interaction with, the tissues of the body. This became obvious from discussions in the literature that described the ideal biomaterial as one that had no effect on blood clotting, or on inflammation and the immune system, and generically was non-cytotoxic. As long as the applications were within simple implantable devices, there was some sense in this, especially when it was appreciated that most materials are modified to some extent by the fluids within the body so that the preferred scenario was one in which there was minimal degradation and minimal response to the material and its degradation products. Over half a century ago, the surgeon's biomaterials armamentarium consisted of a group of such substances. Even then, it soon became appreciated that this was not quite good enough so that the accepted list of biomaterials for long-term implantation was narrowed in order to include only the most degradation resistant materials that the engineering professions could supply, including just a few alloys, based on titanium (Williams, 1977), cobalt-chromium (Metikos-Hukovic et al., 2006) or platinum-palladium (Woodward, 2012), a few oxide ceramics, especially alumina (Webster et al., 1999) and zirconia (Siddiqi et al., 2017), and some thermoplastic or elastomeric materials such as ePTFE (Cassady et al., 2014), acrylics (Frazer et al., 2005), high density polyethylene (Gomez-Barrena et al., 2008), and silicones (Colas and Curtis, 2013).

There is nothing intuitively wrong with this list; it is pragmatic and based on aspects of clinical experience. It could be argued that there was just one biocompatibility specification, and that was the appropriate host response should be no response. The difficulty, which lies at the heart of this paper, is how this empirical list can lead to the development of specifications for the biomaterials of the future. If we take metallic systems as an example, the biocompatibility will be dependent on corrosion rates, which for each alloy system will be dependent on variables such as pH, electrode potential, oxygen potential, galvanic couples and mechanical stress, and on the biological effect of the corrosion products, which will depend on speciation, morphology, stoichiometry, and so on. It is impossible, at this stage, to quantify the risk of adverse host responses in any conceivable system when there are so many independent variables. The same principle applies with all types of biomaterial.

For many years, this problem has been addressed by using surrogates for host responses, and by analogy, biocompatibility. These surrogates largely concern toxicity. As more devices were being developed, regulators became anxious about their decision-making algorithms that had to be based on crude estimates on how biomaterials would perform in the body. This scenario was taken up by standards organizations, to whom regulators looked for guidance on testing procedures. Accordingly, the overarching standards body, ISO, the International Standards Organization, started to produce a series of standard test protocols for the assessment of the biological safety of medical devices, the so-called ISO-10993 series^1^. It is not surprising that this series, now numbering 20 or so parts, in various stages of drafting and revision, has concentrated on this surrogacy, with sections on cytotoxicity, systemic toxicity, genotoxicity, reproductive toxicity, and toxicokinetics of degradation products, included in the recommended tests.

There is some logic to this approach, as manufacturers, clinicians, and regulators should be aware of any toxicological concerns. However, these tests do nothing to further our understanding of biocompatibility pathways and specifications, and should not be considered as the critical determinants of whether specific biomaterials should be used in specific situations.

Most of these tests rely on the effect that extracts derived from the material have on cells in culture. Thus, for cytotoxicity, the material in question is incubated with one of a group of standard extraction media, for example, cotton-seed oil or isotonic saline solution, typically for 72 h at 37°C, and the resulting solution is then incubated with the designated cells, typically fibroblasts, for a further time, again typically 72 h at 37°C, and the effects on the cells noted. With other toxicity and sensitization-type tests, the same type of extraction solution is evaluated either *in vitro* or in a small animal model for short-term effects under standard conditions. In the majority of situations, the results with the text extract are compared to extracts derived from standard, reference, materials.

It will be obvious that such tests have been designed to detect whether any components of a biomaterial that can be leached or extracted from the material in a short space of time can have any negative effect in a designated test system. If there is any component that is not extracted during this 72-h period, its potential effects, either positive of negative, will not be detected. The implications of this are discussed in the next sections. It should be borne in mind that the substances most likely to be extracted will be residual monomers, oligomers, catalysts, and impurities in polymer systems, processing substances in animal tissue-derived biomaterials (such as cross-linking, anti-calcification, and decellularization agents) and surface treatment residues on metallic, and ceramic systems. These are not the substances that control biocompatibility within real-life medical technology situations.

## Implantable Medical Devices

Without doubt, implantable medical devices have played an immense role, over many years, in therapies for a wide spectrum of conditions. Ranging from mechanical support systems for musculoskeletal diseases and trauma, to electronic systems for the control of Parkinson's symptoms and cardiac arrhythmias and to meshes for assisting in complex wound healing processes, millions of patients have been successfully treated with the assistance of such devices. As implied above, the materials used in these devices have predominantly been those of maximal inertness and, generally, provide good long-term performance. However, over several decades there have been many well-publicized situations where devices have not, or appear to have not, provided satisfaction in a significant minority of patients^2^. Of profound importance here is the fact that the materials used in most of these devices will have had previous successful use in implantable devices and will have passed all of the relevant tests for biological safety of the device in question.

It is worth considering briefly what biocompatibility issues have arisen here:

- Silicone breast implants; claims of systemic effects associated with components of the silicone gel, including claims that such components initiate autoimmune conditions such as scleroderma, lupus or rheumatoid arthritis and more recently, anaplastic large-cell lymphoma.
- Silver coated textile sewing rings on mechanical heart valves, with claims of adverse effects on local wound healing,
- Polypropylene urogynecological meshes used for treatment of stress urinary incontinence and pelvic organ prolapse, with claims of degradation-induced adverse local responses, and enhanced susceptibility to infection,
- Metal-on-metal hip replacements, with claims of poor tribological performance, and lymphocytic responses to wear debris,
- Hernia meshes, with claims of adverse host responses to several forms of mesh, either synthetic polymers, or biologically-derived materials.

Since all of these examples have resulted in significant litigation procedures, from which it is difficult to determine the precise details of mechanisms by which the materials could possibly have a role in the causation of adverse host responses, very little unequivocal scientific evidence emerges. This is hugely important since very beneficial, potentially life-saving, or certainly life enhancing, devices may be taken off the market on the basis of a small number of real or imaginary adverse effects that can rarely be proved to be device or material related.

The case of the silver coated heart valves mentioned above is worthy of discussion. For several years, especially in the 1990s, St. Jude Medical (SJM) manufactured several series of mechanical heart valves. In order to try to reduce the risk of bacterial endocarditis with the valves, a design was introduced, on the sewing ring of which was deposited an ultrathin layer of the anti-bacterial metal silver. After undergoing standard preclinical tests, including those for biological safety, the so-called “Silzone” device received regulatory approval in several jurisdictions, including the USA, Canada and Europe, and over 35,000 of the valves were implanted in patients worldwide. After the widely publicized deaths of a few patients in Canada and Wales (UK), claims were made that the silver was implicated in either or both thrombus formation or delayed healing. The concerns of the regulators ensured that the valve was taken-off the market, even though there was no evidence of any causation between silver and such events and in spite of the fact that there were many other potential causes of clinical failure. It was later shown that patients who survived the first few months of implantation (i.e., the vast majority) had equivalent of better long-term performance than similar non-silver coated valves (Grunkenmeier et al., 2006) and that from an actuarial perspective patients who received silver coated SJM valves had survival rates no different to those who had received non-silver valves before the Silzone era or those who received non-silver valves after the Silzone era (Brennan et al., 2015). The one class-action legal case that was resolved, in Toronto, Canada, found in favor of SJM, i.e., there was no proof of causation^3^.

The significance of this in relation to the present paper is that no definitive evidence could be provided to show whether silver could or could not have caused the claimed adverse events. On the one hand it is known that silver does have biological activity (i.e., it is not chemically or biologically “inert” in the context of the definition of biocompatibility discussed above) and the question arises as to whether silver ions were released from the Silzone coating in such a way as to present a risk to mammalian cells in or near the heart. On the other hand, there are many potential causes of heart valve related thrombus formation and delayed healing sufficient to cause a paravalvular leak other than the valve material.

In other words, although the device could, and did, pass standard tests to confirm a low biological safety risk, insufficient was known about the biocompatibility pathways involved with the interaction between silver-coated textiles and the human body so that the risk could be quantified; more importantly, the tests we rely on with respect to biological safety were inadequate to assist in our understanding of these pathways before clinical use was started. This conclusion is valid for any equivalent situation where biological risk is eventually determined to be either positive or negative during clinical use.

In order to assess this conundrum in even more stark terms, it is appropriate to consider the situations with silicone breast implants and autoimmunity and with metal-on-metal hip replacements. In the former example, the major controversy about silicone implants that had such an effect on the implantable device arena in the 1990s eventually centered on the claims that components of silicone gel caused autoimmune diseases. If this were true, it would be of major consequence since these diseases, especially lupus and scleroderma, are clinically very serious. There had never been any previous proof that autoimmunity was caused by any chemical agent but, on the grounds that “absence of proof of harm does not mean that there is no harm” it was initially very difficult to deny that the biocompatibility of silicones could have some autoimmunity-causation component. It was only when several large and very authoritative epidemiological studies showed there was no such link (Janowsky et al., 2000) did the controversy appear to subside (Bondurant et al., 1999). The fact remained, however, that there was no substantial scientific evidence, one way or the other, about the molecular biological characteristics of potential interactions between silicone gel oligomers and the signaling pathways of, for example, scleroderma induction (Al Aranji et al., 2014). There is still no valid test for assessing the risk of biomaterials-induced autoimmunity such that the medical device industry has no definitive answer to the recent resurgence of emotive claims that silicone breast implants have devastating effects on large numbers of women through autoimmunity (Watad et al., 2018). We cannot always hide behind statements that our biomaterials are safe because they pass industry-standard biological safety tests when we do not have clear evidence of the potential biocompatibility pathways. There are no specifications for silicone-based biomaterials that are based on biocompatibility pathways.

There are several differences with respect to metal-on-metal (MOM) hip replacements, but ultimately the problems also arose from a lack of understanding of the relevant biocompatibility pathways. The introduction of new MOM hip prostheses in the early 2000s was based on the perceived need to reduce wear rates in hips in view of the well-documented effect of micron-sized wear particles of acetabular polyethylene components on the macrophage—osteoclast interactions and resulting bone lysis, which caused device loosening (Gallo et al., 2013). It was believed that the significant reduction in the volume of wear particles that would arise if the interface was derived from acetabular and femoral components both made from the same hard alloy would minimize this osteolysis (Allen et al., 2008). While a number of controversial engineering and tribological features contributed to some difficulties that arose (Underwood et al., 2012), the main clinically relevant outcome was a different biocompatibility pathway scenario that was seen with the metal debris. Instead of a preponderance of micron-size particles, which are normally dealt with by macrophage phagocytosis, the bulk of the metal particles were substantially sub-micron (often 10–100 nm) in size (Gill et al., 2012), which could be internalized within lymphocytes, giving, in susceptible patients, an idiosyncratic response of the immune system (Gustafson et al., 2014), often with rapid-onset failure of the device. This was not anticipated on the basis of known metal biocompatibility mechanisms at that time, and could not have been picked up by the standard biological safety tests. Once again, the specifications for these alloys were based on functional characteristics and not on biocompatibility pathways or biological activity.

It should be emphasized that millions of patients, world-wide, are implanted with medical devices, with successful outcomes and no biocompatibility-oriented problems. However, the three scenarios discussed in the previous paragraphs are not unique, and regulatory agencies and manufacturers alike are frequently faced with difficult decisions about whether to allow or keep devices on the market on the basis of a limited number of less-than perfect outcomes that are putatively considered to be caused by poor material selection and resulting toxicity or adverse biological reactions but which could well be due to significant co-morbidities (such as diabetes or obesity) or clinician inexperience.

## Tissue Engineering Products

Tissue engineering has been described as “*the creation of new tissue for the therapeutic reconstruction of the human body, by the deliberate and controlled stimulation of selected target cells through a systematic combination of molecular and mechanical signals*” (Williams, 2006). Clearly these molecular and mechanical signals cannot be effective in a vacuum and some construct will commonly be required to control the relevant processes. These constructs have usually been described as scaffolds. However, the term “template” is preferable since as this implies a different concept; this is so crucial in the next phase of tissue engineering development.

In the area of implantable devices, discussed above, there is already a hint that, without additional agents, biomaterials may not necessarily produce the best results in every situations. Vascular grafts may require endothelial cells in order to generate superior neointima (Meinhart et al., 2005), intravascular stents are assisted by anti-proliferative drugs in the control of in-stent restenosis (Kastrati et al., 2005), spinal fusion devices may be assisted by locally released bone morphogenetic proteins (Lo et al., 2012) and thrombosis of heart valves is inhibited by systemic anticoagulation (Iung et al., 2014). Although the fundamental requirements of the biomaterials remain the same, the achievement of the optimal and appropriate host response is seen to be influenced by biological and pharmacological factors, entirely consistent with the basic tenets of biocompatibility (Williams, 2017).

Taking this argument a little further, if inertness facilitates the biocompatibility of implantable biomaterials, which involves minimal biological activity, how can this be translated into tissue engineering applications where, by definition, the biomaterials have to take part in mechanisms of cell stimulation? A different paradigm is clearly required.

It is of no surprise that the majority of the early group of tissue engineering products to be developed and used in clinical practice incorporated biodegradable polymeric materials that had formed parts of medical devices such as surgical sutures; previous FDA approval in the context of devices constituted the first specification for the new tissue engineering scaffolds. However, a surgical suture was not intended to play a biological role in wound healing; it was simply used to hold tissues together mechanically during healing and then resorb with minimal host response. This was far from the main requirement of a tissue engineering biomaterial, which has to take part in the active process of tissue regeneration.

Considering this from a slightly different perspective, microphotographs of polymeric or ceramic tissue engineering scaffolds usually show that they have been produced by techniques such as solid free form fabrication. The question arises as to whether those microscale porous structures, which should be intended to facilitate the delivery of the “systematic combination of molecular and mechanical signals,” mentioned before, to the target cells, can actually replicate the natural environment of those target cells? In other words, do these structures resemble the niche of the target cells? Furthermore, the niche of these target cells, including stem cells, changes during extracellular matrix expression. If the biomaterial were undergoing degradation and resorption, would its degradation profile be consistent with the profile of cell niche maturation? The answer to these questions is almost certainly no.

It may well be that some tissue engineering processes that involve classical degradable polymers such as poly(glycolic acid) and polycaprolactone do allow the generation of some functional tissue, but this happens in spite of rather than because of the choice of material. More specifically, the tissue-engineering field has progressed in the absence of any clearly delineated specifications for tissue engineering biomaterials or tissue engineering templates. It is now necessary to define these specifications (Williams, 2017); some may be associated with the mechanical characteristics, including those of elasticity (stiffness, compliance etc.), that control the delivery of the necessary mechanical stimuli. Others relate to the delivery of molecular signals and nutrients to the target cells. The majority, however, are concerned with the biocompatibility of these templates, which inevitably will not involve the characteristics of inertness. Essentially, the template has to recapitulate the morphology of the target cell niche and should adapt to the changing microenvironment.

Thus, the tissue engineering biomaterial should have mechanical properties, particularly stiffness, that favor mechanical signaling in order to optimize differentiation, proliferation, and gene expression in the target cells. The material should have surface characteristics that enhance cell adhesion and function and should enable the orchestration of molecular signaling to the relevant cells, through the direction of endogenous molecules and delivery of exogenous molecules. The template should usually be degradable, with relevant degradation kinetics and suitable morphological, and chemical degradation profiles. The material should have a physical form that provides relevant shape to the regenerating tissue and its architecture should optimize the transport of nutrients, gas and biomolecules, either or both *ex vivo* or *in vivo*, and facilitate nerve and blood vessel development. Naturally, the material should be non-cytotoxic, non-immunogenic, and minimally pro-inflammatory.

The concept of replicating the cell niche introduced above is consistent with the trend of recent years. The architecture of tissue engineering templates has been changing, with a move toward hybrid macro- and nano-scale structures and toward hydrogels based on tissues, tissue-derived, or tissue-mimicking components. These include injectable peptide based hydrogels (Greenfield et al., 2010), biomimetic hydrogels (Green et al., 2016), and decellularized tissues (Yu et al., 2016). In such materials, great care has to be taken to avoid undesirable host responses, again consistent with the basic principles of biocompatibility, for example through immunological responses with xenogeneic-derived substances, but this is not the main driving force or specification for their development. Here, the appropriate host response (Williams, 2014) is not no response, but that which is optimal for the stimulation of those target cells within a recognizable, niche-mimicking, microenvironment. Without a clear understanding of the mechanisms of biocompatibility pathways within the tissue engineering context, which may be different to those for long-term implantable devices, there is little chance of designing new functional biomaterials for regenerative medicine.

## Contrast Agents

It is worth mentioning briefly the situation with contrast agents. Anatomical and functional imaging techniques, especially MRI and CT modalities, have been in clinical use for decades, but their utility has been significantly enhanced in recent years through the use of highly specific contrast agents. These are able, for example to accelerate the relaxation times of protons from bulk water in MRI (Peng et al., 2016) or improve targeting ability so that imaging can be used intraoperatively in tumor therapy (McHugh et al., 2018). These contrast agents have typically involved metallic or ceramic nanoparticles or semiconductor quantum dots where issues of *in vivo* distribution, residence time, and toxicity were raised at an early stage.

Until recently, the toxicity of contrast agents has been treated on a case-by-case basis, which has not provided an overall perspective of the potential pathophysiology of conditions that arise from their use. This is perhaps not surprising since they are based on many different chemical structures and morphologies. The situation is made more complex by significant variations in the development of agent-related diseases on the basis of the patient's condition and co-morbidities. For example several gadolinium-based agents have good records of incident free use in MRI but prove remarkably toxic when used in patients with existing renal insufficiency (Ramalho et al., 2016). Individual toxicity profiles exist for contrast agents based on iron oxide (Schmid et al., 2017), gold nanoparticles (Arami et al., 2015), manganese oxides (Pernia Leal et al., 2015), and so on and databases are gradually evolving.

Fortunately, the language of contrast agent biological safety is now turning to concepts of biocompatibility rather than conventional toxicity. This move has been driven by the considerable potential of quantum dots in tumor imaging. Many quantum dot preparations used in non-healthcare applications are based on cadmium compounds, but their recognized cytotoxicity means that clinical applications are highly unlikely (Hardman, 2006). This has provided the opportunity to design quantum dots utilizing metals such as silver or copper, or even carbon, where biocompatibility pathways can be examined and specifications derived from this can be placed alongside functionality specifications in the overall design.

## Biocompatibility Pathways

As noted earlier, the present author has recently discussed mechanisms of biocompatibility in terms of biocompatibility pathways (Williams, 2017). This comprehensive analysis, based on experimental and, especially, clinical evidence, established that the classical views of the development of the host response required reassessment of factors such as the role of protein adsorption on subsequent tissue responses and the temporal sequence of events in inflammation and fibrosis. In particular, in the majority of circumstances the role of protein behavior at biomaterials surfaces is minimal, as is that of surface microtopography; for reviews of the effects of proteins in blood compatibility, see Sefton et al. (2019).

Two major types of mechanism dominate the events in tissues adjacent to biomaterials (which are likely to act synergistically); these are mechanotransduction and sterile inflammation. “Mechanotransduction” describes the processes at the cellular and molecular levels that are involved with the transduction of mechanical stimuli into biochemical signals. Implantable devices do not perform in a stress-free environment, and both structural and hemodynamic forces are likely to be encountered at interfaces. There will, almost inevitably be a mismatch of elastic moduli between tissues and engineering materials; this will result in differential stresses and strains between these components. When forces are applied in normal or abnormal physiological systems, pathways of mechanotransduction which involve sensing and signaling processes, lead to modulation of gene and protein expression profiles, that control sequence of changes in biocompatibility. The timescale will typically be milliseconds/seconds for mechanosensor stretching, hours for modified gene expression and days or weeks with cell function and tissue regeneration. Mechanical forces are inevitably involved in the formation of the response to orthopedic bone and joint replacements, breast implants, vascular grafts, intravascular stents, and many other forms of implanted device. Any discussion of biochemical processes taking place within the host response to a biomaterial has to be superimposed on the effects of mechanical force. Moreover, it does not make sense to assess biocompatibility in a stress-free *in vitro* environment.

Alongside the effects of the mechanical environment are those of the changes of the chemical characteristics associated with the presence of a biomaterial. There are two factors here, the chemical nature of the surface and of any components released from it, and the processes of inflammation, immunity, and fibrosis in the tissues.

It may be possible to demonstrate the effects of modifications to surface chemistries on the release of biological factors from cells *in vitro* but this is rarely relevant to clinical biocompatibility. This is important as literature reviews of biocompatibility may contain citations to this type of *in vitro* study, and these often form the basis of regulatory submissions.

However, some substances are released from these surfaces during contact with tissues by leaching, diffusion, degradation or erosion processes, and when the material is presented to the physiological environment in a labile form. In metallic materials, these may be impurities, metal ions, corrosion products and retained manufacturing, and surface treatment agents. With polymers, they are likely to include monomers and oligomers, catalysts, antioxidants and processing additives, and degradation products. Following decades of clinical experience, the choice of the main biomaterial component in a device has had to be refined; the portfolio of widely accepted engineering materials is now much smaller and is confined to those which are very inert, both biologically and chemically.

When assessing biocompatibility under conditions relevant to *in vivo* applications, we have to take into account these interactions within the context of existing knowledge about mechanisms of sterile inflammation, fibrosis and the response to stress, however they originate. This is based on the immune system; however, the biomaterials community has not been comfortable with the implications of immune system involvement in the host response since the materials are normally considered to be associated with host—non-pathogen interactions, whereas the immune system does address host-pathogen interactions. It is now known that there is a commonalty in the immunological response to danger signals whatever the nature of the stressor. This has given rise to phenomena described as Damage Associated Molecular Patterns (DAMPs), the understanding of which originated with the work of Matzinger (2002) who described the concept of the danger model, replacing the standard self and non-self paradigm. Sterile inflammation was described as inflammation that results from trauma or chemically-induced injury without the involvement of any microorganism. It is associated with the recruitment of cells such as neutrophils and macrophages and the generation of pro-inflammatory chemokines and cytokines, especially IL-1 and TNF. With respect to biomaterials, including those used in devices that have an extended residence time in the body, the progress of biomaterial-induced sterile inflammation throughout this period has to be considered; it is helpful to note that this involves mechanisms similar to sterile inflammatory diseases, which may be associated with both endogenous and exogenous substances; examples include gout and pneumoconiosis.

There are several processes that are mechanistically involved in the sterile inflammatory process (Chen and Nunez, 2010). Importantly, pattern recognition receptors (PRRs) on inflammatory cells, which can sense conserved structural entities in microorganisms, also sense some exogenous molecules. The released intracellular cytokines and chemokines activate common pathways downstream, where innate multiprotein complexes, the inflammasomes, induce inflammation in response to both pathogens and molecules derived from the proteins of the host. It is also noted that in all types of fibrosis, whatever the cause, inflammatory-immunologic reactions take place that upregulate pro-fibrotic processes; fibrosis around an implant occurs simultaneously with inflammation and is not a separate event.

It should be obvious here that the characteristics of the stress environment control those features of the inflammation-fibrosis response, which influence the eventual outcome and identification of the pathways that are associated with both sterile inflammation and mechanotransduction will facilitate this understanding. This approach to the host response also subsumes the role of macrophages, where evidence now points to processes whereby these cells undergo time-dependent phenotypic and functional alterations according to the stress factors. These lead to either pro-inflammatory or anti-inflammatory situations (Wynn and Vannella, 2016), that are dependent on the DAMP profiles.

This discussion has focused on the innate immune response. However, other components of immune responses, and also other forms of toxicity, may be involved, possibly explaining some of the difficult clinical biocompatibility events, especially those of idiosyncratic feature, including adaptive immune responses (especially hypersensitivity), autoimmune effects, and genotoxicity. It could also be instructive to use models of adverse outcomes pathways that have recently been developed within general toxicology, for example as used by Zhang et al. with respect to the comparative toxicity of contrast agents (Zhang et al., 2018).

## Control of Biocompatibility Through Modulation of Biocompatibility Pathways

The above analysis shows that it should be possible to influence biocompatibility characteristics through a modulation of biocompatibility pathways, possibly by locally delivered pharmaceutical agents or by control of biomaterial architecture or morphology. There is little consistent data on these possibilities and it is not yet possible to create generic paradigms. However, a few examples of the way forward can be quoted.

With respect to mechanotransduction, several recent studies have pointed to some key pathways. For example, Janson and Putnam (2015) have highlighted the signaling pathways that have been implicated in mechanotransduction through the effects of topographical cues; cells share common mechanisms to respond to physical and chemical topography and to matrix elasticity, potentially leading to changes in gene transcription. Molecular components here include integrins, focal adhesion-associated proteins such as FAK, and the RhoA/ROCK/MAPK axis. Lee et al. have similarly demonstrated the role of nuclear mechanosensitivity in determining cellular responses, such as the way in which matrix stiffness alters laminin A/C expression in mesenchymal stem cells, which ultimately determines the lineage specification (Lee et al., 2019).

With respect to inflammatory responses, Liu et al. have shown that the size of graphene oxide particles influences phagocytosis processes through modulation of interactions with toll-like receptors and activation of NF- κB pathways (Ma et al., 2015). Inflammatory responses to cobalt-chromium dental alloys have been shown to be due to upregulation of pro-inflammatory cytokines such as TNF- α, IL-1 β, IL-6, and IL-8. The alloys activate the NRF2 pathway, up-regulate antioxidant enzymes including heme oxidase-1 and activate JAK2/STAT3, p38/ERK/JNK MAPKs, and NF- κB pathways (Kim et al., 2016).

The classical foreign body response may now be seen as a process regulated by specific biochemical pathways, the nature of which will depend on the local circumstances. Liao et al. have shown that the response to the widely used implant material polyetheretherketone is controlled by the miR-29ab1-mediated SLT3 upregulation, and that this may be influenced by the local delivery of pravastatin (Liao et al., 2014). With degradable magnesium alloys, the stimulation of osteogenesis may be achieved *via* the upregulation of transcription factors in the ERK signaling pathway through the effects of released metal ions such as calcium and strontium (Li et al., 2016); the overall effects of magnesium on the fate of mesenchymal stem cells mediated *via* different pathways has been reviewed by Luthringer and Willumeit-Romer (2016). Huang et al. have recently shown that silicon, magnesium and calcium ions released from silicate bioceramics inhibit macrophage inflammatory responses by suppressing the activated inflammatory MAPK and NF- κB pathways (Huang et al., 2018), while Pang et al. have similarly reported the effects of different modified hydroxyapatite structures on the expression of both inflammatory and anti-inflammatory cytokines (Pang et al., 2019). The possibility of modifying the activation of the ERK1/2 signaling pathway during osteogenic differentiation of mesenchymal stem cells through various functional groups including -OH, -COOH, -NH_2_, and -CH_3_ has been shown by Bai et al. (2013). Wang et al. have shown that chitosan-collagen composite films can regulate the expression of osteoblastic marker genes, and specifically that osteoblast differentiation of mesenchymal stem cells can be promoted through an ERK1/2 activated Runx2 pathway (Wang et al., 2016).

With nanoparticles, the macrophage inflammatory activity of titania nanotubes is attenuated if he MAPK and NF-κB pathways are inhibited (Neacsu et al., 2015) and gold nanoparticles promote the differentiation of embryonic stem cells into dopaminergic neurons *via* the mTOR/p70S6K pathway (Wei et al., 2017) or the osteogenic differentiation of periodontal ligament cells *via* the p38 MAPK pathway (Niu et al., 2018). Poly(lactic acid) nanoparticles are internalized in lung epithelial cells through clathrin-coated pits and lipid rafts (Da Luz et al., 2017) while the effect of PAMAM dendrimers on the activation of pro-inflammatory signaling pathways, especially involving NF-κB transduction may be influenced by pyrrolidone modification (Janaszewska et al., 2017). Pathways for nanoparticle-induced apoptosis with cerium oxide (Khan et al., 2017) and autophagy with silver (Mao et al., 2016) have been identified.

The examples given in the preceding paragraphs merely give a hint of how the identification of pathways associated with biocompatibility phenomena, ranging from fibrotic and inflammatory responses to implanted materials to stem cell differentiation associated with biomaterial templates and nanoparticle toxicity, can lead to the modulation of these phenomena and the potential optimization of biocompatibility performance.

## Conclusions

The principal conclusion of this perspectives paper on the fundamental characteristics of biocompatibility is that, through a far better understanding of the precise mechanisms of biocompatibility phenomena, and especially the biological pathways that are involved, it should be possible to influence these phenomena, such modulation improving the clinical outcomes associated with biomaterials. During recent years, significant progress has been made with identifying the specific mechanisms, especially those of mechanotransduction and sterile inflammation, that should now profoundly modify the classical concepts of the foreign body response, allowing, through very different objectives, the optimization of biocompatibility outcomes with implanted devices, tissue engineering templates, imaging contrast agents, and other biomaterials-based technologies. The control of biocompatibility, rather than the simple subjective observation of events, should significantly improve biomaterials performance.

## Author Contributions

The author confirms being the sole contributor of this work and has approved it for publication.

### Conflict of Interest

DW is a Professor at Wake Forest Institute of Regenerative Medicine. He is also Founding Director and Chairman of Strait Access Technologies (Pty) Ltd., South Africa. The work performed for this paper was conducted in the absence of any commercial or financial relationships that could be construed as a potential conflict of interest.

## Footnotes

^1^International Standards Organization, ISO 10993-1 Biological Evaluation of Medical Devices—Part 1: Evaluation and Testing Within a Risk Management System, Revised 2018.

^2^17 Food and Drugs Administration: Statement from FDA Commissioner Scott Gottlieb MD and Jeff Shuren MD, Director of the Center for Devices and Radiological Health, on efforts to evaluate materials in medical devices to address potential safety issues. March 15th 2019.

^3^Andersen v. St. Jude Medical, Inc. (2012). ONSC 3660 COURT FILE NO.: 00-CV- 195906CV, Toronto, CA.
